# Update on the Diagnosis and Management of Meningiomas

**DOI:** 10.3390/cancers15143575

**Published:** 2023-07-12

**Authors:** Francesco Maiuri, Marialaura Del Basso de Caro

**Affiliations:** 1 Department of Neurosciences and Reproductive and Odontostomatological Sciences, Neurosurgical Clinic, 80131 Naples, Italy; 2 Department of Advanced Biomedical Sciences, Section of Pathology, School of Medicine, University “Federico II” of Naples, 80131 Naples, Italy

This series of five articles (one original article and four reviews) focuses on the most recent and interesting research studies on the biomolecular and radiological diagnosis and the surgical and medical management of meningiomas.

The WHO Classification of 2021 [1] defines the criteria for inclusion into the three histological grades (I, II, III) and identifies 15 different meningioma subtypes. However, it is well known in clinical practice that meningiomas of the same histological grade, mainly grade I meningiomas, show different biological and clinical behavior; this is the case for grade I meningiomas with aggressive behavior and early recurrence.

Many studies on the molecular aspect of meningiomas have disclosed several molecular alterations [2,3,4], most frequently the loss of the neurofibromin 2 (NF2) gene on chromosome 22 [5,6] and TERT promoter mutations [7,8]. The inclusion of these biomolecular markers in diagnostic assessment may allow the identification of patients with a higher risk of progression or recurrence who require close follow-up imaging studies and more aggressive treatment.

Magnetic resonance imaging (MRI) is the modality of choice for assessing meningiomas. In recent years, radiomics applications have been shown to provide additional information. Radiomics is an emerging technique which collects and analyzes high-dimensional quantitative features derived from an explored region. The radiomics process starts with image acquisition and preprocessing, followed by lesion segmentation; then, the extraction of reliable features can be performed. This technique allows for the correlation of quantifiable images of heterogeneous areas within a lesion with previously established pathological and genotypic characteristics. In this way, it is possible to detect tumor features that cannot be identified through traditional analyses [9,10,11,12,13,14,15]. Although radiomics programs for tumor segmentation and characterization are commercially available, those providing predictive and prognostic assessment are not still available in clinical practice. However, radiomics must be considered a valid technique with significant development possibilities. 

Although the surgical management of meningiomas is well codified, several still-controversial aspects remain, such as how to define the extent of tumor resection, how to manage invasive meningiomas (aggressive versus more conservative resection) and when to use the endonasal transbasal approaches for midline skull base meningiomas. The Simpson classification, published in 1957 [16], is still used for grading the extent of meningioma resection. However, it was introduced in the pre-microsurgical era; additionally, the evaluation of residual tumor in grade IV is subjective and does not consider the meningioma location. For these reasons, many studies, mainly those in the last 10 years [17,18,19,20,21,22,23], have questioned the Simpson classification and its value in predicting meningioma recurrence. Of the utmost importance is defining the size of tumor remnants in grade IV resections, from small tissue tumor remnants left on the cortex to significant residual nodules. Thus, the Simpson classification is today insufficient and should be modified according to the data of postoperative MRI studies that assess the gross total versus subtotal resection and quantify the size of the residual tumor. Although complete tumor resection with resection or wide coagulation of the dural attachment is the goal of meningioma surgery, this is sometimes difficult or even impossible for some invasive meningiomas. In these instances, an aggressive resection must be balanced with the risk of injury to the neurological and vascular structures [19]. Thus, when complete resection cannot be achieved, it is advisable that the residual tumor be reduced as much as possible, with the aim of increasing the effect of the postoperative radiosurgery. In fact, it has been shown that Simpson grade II and III resections show similar recurrence-free survival rates compared to grade IV recession with radiotherapy [21].

Skull base meningiomas have always represented a challenge for neurosurgeons due to their proximity to important nervous and vascular structures. They have traditionally been operated on through transcranial approaches, which carry the risk of brain damage. In recent decades, midline skull base meningiomas have been treated with increasing frequency through endoscopic endonasal approaches [24], which prevent brain retraction and obtain safe maximal resection with better clinical outcomes. Tuberculum sellae meningiomas grant access to the tumor attachment and vascular feeders [25,26,27]. Selected cases of olfactory groove meningiomas [28,29,30,31] and clivus meningiomas [32,33] may also be treated through extended endoscopic endonasal approaches, while large-size tumors with a hard consistency and close proximity to the vascular structures are contraindications to transabasal approaches. Postoperative cerebrospinal fluid leak is the main surgical problem. The recurrence rates of endoscopic basal approaches will better be defined in the next few years after studies with a longer follow-up period are conducted.

Medical therapy for more aggressive and recurrent meningiomas is still limited in its efficacy, although different cytotoxic agents have been used. In the last decade, the identification of molecular alterations in more aggressive meningiomas has suggested the need for a biomolecular classification with the aim of defining tailored medical treatments [34,35,36]. However, their efficacy must be confirmed by larger studies in the next few years.
